# High rate of transplacental infection and transmission of *Neospora caninum* following experimental challenge of cattle at day 210 of gestation

**DOI:** 10.1186/1297-9716-43-83

**Published:** 2012-12-10

**Authors:** Julio Benavides, Frank Katzer, Stephen W Maley, Paul M Bartley, Germán Cantón, Javier Palarea-Albaladejo, Caroline A Purslow, Yvonne Pang, Mara S Rocchi, Francesca Chianini, David Buxton, Elisabeth A Innes

**Affiliations:** 1 Moredun Research Institute, Pentlands Science Park, Bush Loan, Edinburgh, EH26 0PZ, United Kingdom; 2 Instituto Nacional de Tecnología Agropecuaria (INTA), EEA Balcarce, CC276, Argentina; 3 Biomathematics & Statistics Scotland, The King's Buildings, Edinburgh, EH9 3JZ, Scotland, United Kingdom; 4 Present address: Instituto de Ganadería de Montaña, (CSIC-ULE), 24346, Grulleros (León), Spain

## Abstract

In order to investigate the pathogenesis of neosporosis following a primary infection in late pregnancy, cattle were subcutaneously challenged with 5 × 10^8^*Neospora caninum* (NC1 isolate) tachyzoites at day 210 of gestation and serial necropsies were then carried out at 14, 28, 42 and 56 days post-infection (dpi). No abortions occurred and all the foetuses were viable at the time of euthanasia. There was a high rate of vertical transmission, as parasites were detected by immunohistochemical labelling and PCR in all the foetuses from 28 dpi. Focal necrotic lesions were observed in the placentomes of the placenta from 28 dpi and showed resolution during later time points, denoted by infiltration of inflammatory cells at 42 dpi and fibrosis at 56 dpi. Foetuses at 28 and 42 dpi showed scarce and isolated lesions which are unlikely to represent a threat to foetal viability. No lesions were observed in the foetuses at 14 or 56 dpi suggesting control of the infection and resolution of the lesions by maternal and foetal immune responses. Once infection was established, it could not be cleared from the host and vertical transmission of the parasite occurred in all infected hosts. Parasite was detected in the placenta at 28 dpi, while in previous experimental infections of cattle at day 70 and 140 of gestation using the same challenge model, it was already present at day 14 post infection. This suggests that a change in the maternal immune response plays a crucial role in limiting the initial infection during the last term of pregnancy.

## Introduction

*Neospora caninum* is a cyst-forming protozoan parasite causing major reproductive losses in cattle and sporadic neurological disease in dogs. This parasite shows a heteroxenous life cycle, with dogs and cattle acting as the main definitive and intermediate hosts, respectively (reviewed by Dubey et al. [1]). Cattle can become horizontally infected, through the ingestion of sporulated oocysts shed in the faeces of infected dogs, or by vertical transmission, when infection is transplacentally transmitted from the pregnant mother to the foetus. Vertical transmission may occur when the mother is infected prior to pregnancy, following recrudescence of the parasite infection (endogenous transplacental infection), or when the mother becomes infected during pregnancy (exogenous transplacental infection) [2]. Both endogenous and exogenous vertical transmission, are associated with the occurrence of abortions; however they represent different epidemiological phenomena, with the endogenous transplacental infection occurring more frequently than exogenous transmission [3]. Vertical transmission is the most important way of transmission of the parasite and maintaining it within the cattle population. It has been demonstrated that *N. caninum* is a major cause of abortion worldwide and therefore has very serious welfare and economic consequences [4,5].

Infection of the host usually leads to the formation of tissue cysts, predominantly in neural tissues, allowing the parasite to persist in infected animals, although sometimes, the infection or re-activation from a latent state, leads to abortion (reviewed by Innes et al. [6]). The pathogenesis of these abortions is not yet fully understood and the precise mechanisms involved in vertical transmission to the foetus remain mostly unknown (reviewed by Dubey et al. [1]). However, there is clear experimental evidence that the time of gestation when the infection and parasitaemia occurs is a key component in the pathogenesis of the disease [6,7]. Infection during early pregnancy, i.e. day 70 of gestation is associated with a high rate of foetal death and resorption whereas infection later in pregnancy, i.e. beyond day 140 of gestation, generally results in congenitally infected foetuses, born alive and usually with no clinical signs of infection [6-11].

Maternal immune responses are important in controlling bovine neosporosis, as it has been shown that infected cattle elicit a Th1 type response, based on CD4-lymphocyte activation and gamma interferon (IFN-γ) production [7,12,13], which is effective in controlling the multiplication of the parasite [14]. IFN-γ production during pregnancy is effective in preventing abortion in naturally infected cows [15,16]. Although required to control the parasite, the triggering of this Th1 type response in the placenta may be detrimental to the foetus and it has been considered to be a possible cause of abortion associated with infection during the first trimester of the pregnancy [12,17,18]. In order to maintain the pregnancy, as it progresses, there is a cytokine regulation (immunomodulation) of the maternal immune response at the placenta to counteract any pro-inflammatory response [19], because a Th1-type immune response is considered incompatible with pregnancy in mice and humans [20]. Recent data suggests that a pro-inflammatory response is part of the physiology at some stages of successful pregnancies (reviewed by Chaouat [21]), and it has been found that an active maternal immune response in the placenta, formed by both pro-inflammatory and anti-inflammatory cytokines, is beneficial for limiting the consequences of *N. caninum* infection during pregnancy [22,23]. The immune response during pregnancy is very complex, and it has been shown that cattle infected with *N. caninum* at mid gestation elicit a significantly decreased peripheral antigen-specific Th1-type response when compared to infection pre-pregnancy or at early gestation. This immunomodulation may contribute to maintenance of the pregnancy, at the expense of controlling the parasite and therefore allowing vertical transmission of *N. caninum* to the placenta and foetus [24].

Another critical factor in bovine neosporosis, which also evolves during pregnancy, is the immune response of the foetus [17,25]. Foetal immuno-competence begins to develop at around day 80 of gestation [26] and foetuses are able to mount a parasite specific humoral response against *Neospora* at least from day 100 of gestation onwards [27]. This progressive maturation of the foetal immune system is reflected in the absence or reduction of inflammatory lesions, whilst widespread dissemination of the parasite and *N. caninum* specific necrotic lesions are evident in early pregnancy [10,28]. Infection in mid or late pregnancy results in a non-purulent inflammatory infiltrate, fewer lesions and limited parasite distribution in the foetus [8,11,28]. This observation has led some authors to suggest that it is the immunological maturity of the foetus that controls the infection, rather than the maternal inflammatory response at the placenta, that determines the survival of the foetus or the occurrence of an abortion [28].

The timing of infection during pregnancy is a pivotal factor in the pathogenesis of bovine neosporosis, as demonstrated by analysis of the pathogenesis and maternal and foetal immune responses to experimental *N. caninum* infections during early and mid-gestation [10-12,27,29]. The present study aims to examine the pathogenesis of a primary infection with *N. caninum* at day 210 of gestation in cattle using similar challenge and examination methodologies to those used in previous experimental challenges at days 70 and 140 of gestation [10-12,27,29]. This will allow direct comparison of parasitaemia, distribution of the parasite, lesions and pathogenesis in foetus, placenta and dams to the results from the previous experiments.

## Material and methods

### Animals and experimental design

Twenty-one Aberdeen Angus cross or Belgian Blue cross cattle aged 20 to 23 months, seronegative for *N. caninum*, *Toxoplasma gondii*, bovine viral diarrhoea virus, infectious bovine rhinotracheitis and *Leptospira hardjo* were oestrus synchronized and artificially inseminated with a mixture of semen from different bulls as previously described [11]. Pregnancy and foetal viability were confirmed by ultrasound scanning on day 35 after insemination and fifteen pregnant cows were selected for the experiment.

Animals were observed twice daily throughout the experiment. Rectal temperatures were recorded two days before inoculation and then daily until 14 dpi. Animals were considered to be febrile when the temperature was over 39.5°C. Belgian Blue cross animals were randomly allocated into the infected sub-group using a random number generator. The remaining animals (two Aberdeen Angus cross and two Belgian Blue cross) were allocated into the control group (see Additional file 1). All animals were housed together until the end of the study. At day 210 of gestation animals (at 27 to 30 month of age) were inoculated (see below) Three infected animals and one uninfected-control animal were culled at 14, 28 and 42 days post-inoculation (dpi) by intravenous barbiturate overdose (Table 1), following which, dams and foetuses were examined at post-mortem. At 56 dpi, the remaining animals were culled and examined at post-mortem.

**Table 1:** Results of PCR parasite detection in blood samples taken from infected and uninfected-control dams from −1 to 14 dpi

**Animal ref.**	**dpi**															
	−1	0	1	2	3	4	5	6	7	8	9	10	11	12	13	14
A	**-**	**-**	**-**	**-**	**-**	**-**	**-**	**-**	**-**	**-**	**+**	**-**	**+**	**-**	**-**	**-**
B	**-**	**-**	**-**	**-**	**-**	**-**	**-**	**-**	**-**	**+**	**-**	**+**	**-**	**-**	**+**	**-**
C	**-**	**-**	**-**	**-**	**-**	**-**	**-**	**-**	**-**	**+**	**-**	**-**	**-**	**-**	**-**	**-**
D	**-**	**-**	**-**	**-**	**-**	**-**	**-**	**-**	**-**	**-**	**-**	**-**	**+**	**-**	**-**	**+**
E	**-**	**-**	**-**	**-**	**-**	**-**	**-**	**-**	**-**	**-**	**+**	**+**	**-**	**-**	**+**	**+**
F	**-**	**-**	**-**	**-**	**-**	**-**	**-**	**-**	**-**	**-**	**-**	**-**	**+**	**-**	**-**	**-**
G	**-**	**-**	**-**	**-**	**-**	**-**	**-**	**-**	**-**	**-**	**+**	**-**	**+**	**-**	**-**	**-**
H	**-**	**-**	**-**	**-**	**-**	**-**	**-**	**-**	**-**	**-**	**+**	**-**	**-**	**-**	**-**	**-**
I	**-**	**-**	**-**	**-**	**-**	**-**	**-**	**-**	**-**	**-**	**-**	**-**	**+**	**+**	**-**	**-**
J	**-**	**-**	**-**	**-**	**-**	**-**	**-**	**-**	**-**	**-**	**-**	**+**	**-**	**+**	**-**	**+**
K	**-**	**-**	**-**	**-**	**-**	**-**	**-**	**-**	**-**	**-**	**-**	**-**	**-**	**+**	**+**	**-**
Uninfected-Controls																
(Animals L, M, N and O)*	**-**	**-**	**-**	**-**	**-**	**-**	**-**	**-**	**-**	**-**	**-**	**-**	**-**	**-**	**-**	**-**

* As all control animals (n = 4) were PCR negative at all times point tested, they have been grouped.

Blood samples were collected at regular intervals to monitor humoral and cell-mediated immune responses (Bartley et al., in preparation).

All animal procedures complied with the Animals (Scientific Procedures) Act 1986 and were approved by the Moredun Research Institute ethics committee.

### Experimental inocula

Animals from the infected group were each subcutaneously inoculated over the left prefemoral lymph node with 2 mL of PBS containing 5 × 10^8^ live *N. caninum* tachyzoites (NC1 isolate) [30]. The tachyzoites were cultured in Vero cells and the inocula consisted of the same low-passage (p-36) stabilate, which was cryopreserved at −180°C in vapour phase liquid nitrogen storage, since the early and mid gestation experiments in pregnant cattle [10,11]. Animals from the uninfected-control group were each inoculated with 5 × 10^7^ Vero cells, which is equivalent to that found in the parasite inocula, in 2 mL of PBS.

### Collection of tissue samples for histopathology and ITS1 PCR

Post-mortem examinations of the dams and foetuses were carried out immediately after euthanasia and tissue samples were taken and placed into 10% formal saline. From each placenta, ten randomly selected placentomes were chosen and sliced coronally. Maternal samples included brain, apex of the heart, lung, liver, spleen, kidney, mammary gland, skeletal muscle (semitendinosus, diaphragm and psoas major) and lymph nodes (medial retropharyngeal, left and right iliofemoral (uterine) and left and right subiliac (prefemoral)). Foetal tissue samples included brain, spinal cord, left eye, apex of the heart, lung, liver, kidney, thymus, semitendinosus muscle and lymph nodes (hepatic, medial retropharyngeal and caudal mesenteric). After fixation for five days, maternal and foetal brains were cut coronally from frontal cerebrum to medulla, to include 11 different areas, and processed, with the rest of the samples, by standard procedures for haematoxylin and eosin staining [11]. Selected sections from the placentomes were stained with MSB (Martius, Scarlet and Blue) for the detection of fibrin and with Gomori trichrome stain to show connective tissue. Samples of the same maternal, placental and foetal tissues were collected for DNA extraction. These samples were stored at −20°C prior to analysis.

### Immunohistochemistry

Wax sections were cut from the formalin-fixed foetal, placental and maternal tissues and were immunolabelled for *N. caninum* antigens using a polyclonal serum, raised against *N. caninum*, according to previously described methods [11].

### Extraction of *Neospora* DNA from blood and tissue samples

Samples of heparinised blood were taken daily by jugular venupuncture from the day before the inoculation until 14 dpi. After sampling, a 3 mL aliquot of whole blood was mixed for 15 min at room temperature with freshly made 9 mL of red blood cell lysis buffer (144 mM NH_4_Cl, 170 mM Tris HCl pH 7.65), followed by centrifugation at 1000 *g* for 15 min to pellet white blood cells and free parasites. The pellet was washed one more time in 5 mL red blood cell lysis buffer, resuspended in 200 μL PBS followed by lysis with Proteinase K (0.1 mg per mL, final concentration) and storage at −20°C prior to ITS1 PCR analysis. Samples of tissue (approximately 1 g) were defrosted, finely chopped and transferred to a Precellys tissue homogeniser tube containing 1 mL nuclei lysis solution (Promega) and homogenised for 2 × 50 s at 6500 rpm (Precellys 24 tissue homogeniser). A 400 μL aliquot of the resultant homogenate was processed to DNA using the Wizard® genomic DNA purification protocol (Promega). The DNA was resuspended in 200 μL of DNase/RNase free water and stored at −20°C prior to ITS1 PCR analysis.

### Detection of *Neospora* DNA using *N. caninum* specific nested ITS1 PCR

The nested ITS1 primers NN1 – NN2 and NP1 – NP2 were previously described by Buxton et al. [31]. The reaction mixture and amplification conditions were as follows. Each reaction (20 μL) consisted of 2 μL of 10× custom PCR master mix, giving a working concentration of 45 mM Tris–HCl pH 8.8, 11 mM (NH_4_)_2_SO_4_, 4.5 mM MgCl_2_, 0.113 mg/mL BSA, 4.4 μM EDTA and 1.0 mM each of dATP, dCTP, dGTP and dTTP (ABgene, Surrey, UK), 1 μL each forward and reverse primer (5pmol) (primary amplification- NN1, NN2; secondary amplification- NP1, NP2), 0.15 μL (5 U/μL) *Taq* polymerase (Bioline Ltd. London, UK), 13.85 μL DNase / RNase free water and 2 μL sample DNA, with each sample being analysed in triplicate. The reaction conditions for both primary and secondary amplifications were as follows 95°C for 5 min followed by 35 cycles at 95°C for 1 min, 55°C for 1 min and 72°C for 1 min and a final extension period of 72°C for 5 min. The primary amplicon was diluted with 100 μL DNase / RNase free water and 2 μL of the diluted product was used as template DNA for the second round amplification. Following which, 10 μL of second round product was analysed by 2% agarose gel electrophoresis, stained with GelRed™ (Biotium Inc., Hayward, USA) and visualised by UV light. A sample was considered positive if one of the triplicates gave visible band of 279 bps.

### Satellite marker analysis

Nested PCR, using the same reaction conditions as for the ITS1 PCR, were conducted for a *N. caninum* specific satellite marker (MRI42). The primers used in the first round PCR were MRI42Fo (acacggaagagagcctagca) and MRI42Ro (ttcgttcacggacaagacac). The reactions from the first round PCRs were diluted with 100 μL DNase / RNase free water and 2 μL of the diluted product was used as template DNA for the second round amplification. The second round primers were MRI42Fi (gcattcacttttgtccgtgt) and MRI42Ri (aacaggaatgcctccaactg). Following which, 10 μL of second round product was analysed by 3% MetaSieve agarose (Flowgen-Bioscience Ltd, Hessle, UK) gel electrophoresis, stained with GelRed™ and visualised by UV light. The satellite region consisted of 36.5 repeats of ATAG and 17.5 repeats of TA located on chromosome 1 b (Contig 1004) from position 1662628 to 1662868 generating a 241 bp amplicon for the NCLiv isolate.

### Statistical analysis

Rectal temperature data between 1 and 14 dpi were modelled using a linear mixed model with a normal error structure fitted by REML. Treatment (infected/control animal), time, treatment-by time interaction and baseline temperature (mean temperature over two days before inoculation) were regarded as fixed effects, whereas animal effects were introduced as random. A repeated measures correlation pattern was incorporated as a first order autoregressive model. Model selection was based on AIC and likelihood ratio tests. Statistically significant terms were determined at the usual level of *p* = 0.05.

## Results

### Clinical observations

**Figure 1:**
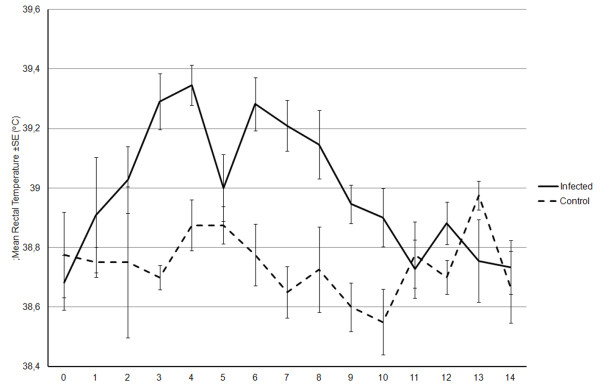
**Mean maternal rectal temperature following *N. caninum *tachyzoite challenge.** Mean maternal rectal temperatures (± standard error bars) over time for infected cattle (solid line) and for uninfected-control cattle (broken line). The infected group showed two peaks of fever at about days 4 and 6 post challenge. Mean temperatures of control animals remained under 39.0° for the whole experiment.

On the day of euthanasia, all dams carried a viable foetus. Overall statistically significant differences in mean temperature response were found between infected and uninfected-control groups (*p* < 0.001). The mean rectal temperature of the infected group remained higher than that of the control group from 2 until 10 dpi. There was an initial mean temperature peak at around 3–4 dpi, 39.3°C at 4 dpi, followed by a fall of the mean temperature to 39.0°C on 5 dpi and a second increase to a mean of almost 39.3°C on 6 dpi. Mean temperatures returned to control levels on 9 dpi (Figure 1). Among the infected group, 8 out of 11 animals showed fever at some point between 1 and 8 dpi, and one of those animals was also febrile on 13 dpi. The mean rectal temperature in the uninfected-control group remained at or below 39.0°C throughout the monitoring period. The effect of age (at the time of challenge) and breed of the dams on the febrile responses from both the infected and control groups is illustrated in Additional file 1.

### Parasitaemia

Using ITS1 PCR, parasite DNA was detected in blood samples from all infected dams at least once between 8 and 14 dpi. Positive PCR results were obtained at more than one time point for 9 out of 11 infected dams. No parasite DNA was detectable in blood samples from the uninfected-control animals at any of the time points (Table 1).

### Gross pathology

No gross lesions were observed in any of the dams, placentas or foetuses, either from the infected group or the uninfected-control group, during the post mortem studies.

### Histopathology and immunohistochemestry

#### Placental tissues

**Figure 2:**
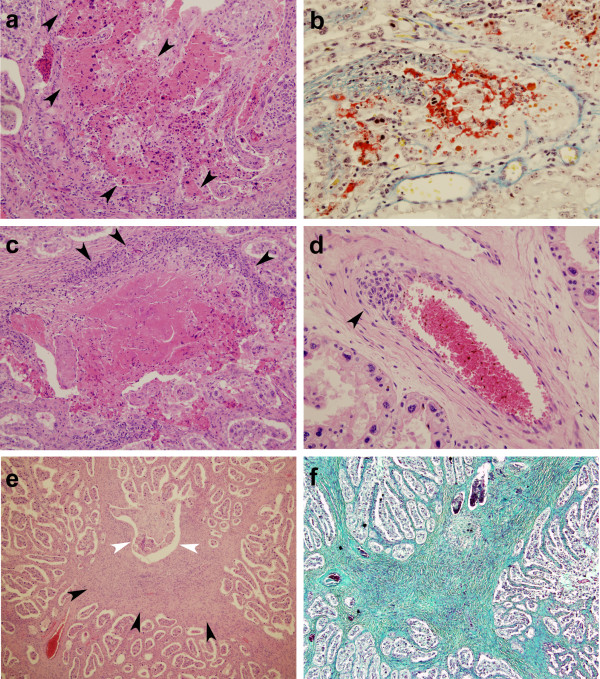
***Neospora caninum *specific placental lesions following subcutaneous challenge of pregnant cattle at day 210 of gestation. a**: 28 dpi. A focus of necrosis (arrowheads), mainly affecting foetal mesenchyme but involving also adjacent areas of maternal caruncular septa. HE. ×100. **b**: 28 dpi. Serum leakage and formation of fibrin (red) is showed between the foetal villus (right) and the maternal septa (left). MSB. ×200. **c**: 42 dpi. Focus of necrosis affecting both maternal and foetal tissue. There is a marked mononuclear cell inflammation in the periphery of the focus (arrowheads). HE. ×100. **d**: 42 dpi. Focus of mononuclear inflammation (arrowhead) at the tunica intima and media of an arteriole located at the maternal caruncular septa. HE. ×200. **e**: 56 dpi. Prominent thickening and hypercellularity of the maternal caruncular septa (arrowheads). Atrophy and loss of trophectoderm of foetal villi adjacent to the thickened area (white arrowheads). HE. ×40. **f**: 56 dpi. Proliferation of connective tissue (green) in a thickened area of the maternal caruncular septa. Gomori Trichrome. ×40.

**Figure 3:**
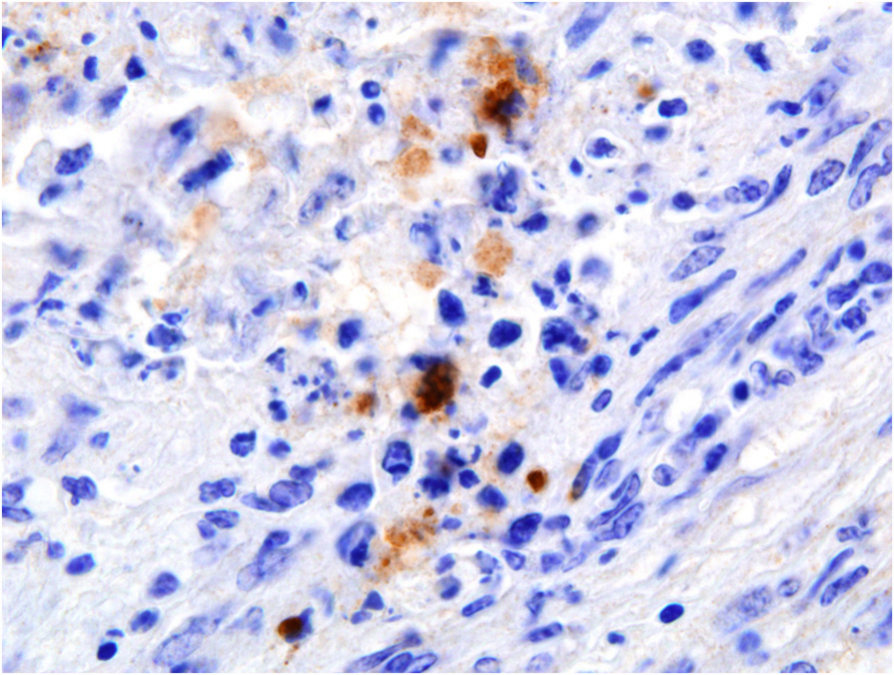
**Immunohistochemical labelling of *Neospora *antigen in a placentome.** Section of placentome collected at 28 dpi, showing immunolabelled *N. caninum* tachyzoite-like structures (brown) in the maternal caruncular septa in small focal area of necrosis. HE. ×200.

No lesions were observed at 14 dpi. At 28 dpi, the placentas from two of the three infected animals showed focal lesions in six and seven out of ten placentomes studied in each of these animals (Table 2). These lesions were characterized by foci of coagulative necrosis or cytolysis of foetal villi accompanied by necrotic debris and extravasation of a proteinaceous eosinophilic exudate into the space between the affected villus and the maternal caruncular septum (Figure 2a). This proteinaceous material was shown to contain variable amounts of fibrin using the MSB stain (Figure 2b). The sizes of the necrotic foci were variable, although usually they were formed by the coalescence of several degenerated villi, and usually only one, never more than two, were seen per slide. Infrequent accumulations of mononuclear inflammatory cells were present in the adjacent caruncular septa in few placentomes. *Neospora-*antigen was labelled by immunohistochemistry as tachyzoite-like structures located at necrotic lesions at the caruncular septa (Figure 3). The presence of the parasite was detected in two placentas out of the three studied and in two placentomes per placenta (Table 2).

**Table 2:** Parasite detection and histological changes in placenta, foetuses and dams from infected animals

**dpi**	**Animal ref.**	**Results in studied samples**		
		**Placenta**	**Foetus**	**Maternal**
		**PCR/HE/IHC**	**PCR/HE/IHC**	**PCR/HE/IHC**
14	A	−/−/−	−/−/−	−/−/−
	B	−/−/−	−/−/−	−/−/−
	C	−/−/−	−/−/−	−/−/−
28	D	+/−/−	−/+/+	−/−/−
	E	+/+/+	+/+/+	+/−/−
	F	+/+/+	+/+/−	−/−/−
42	G	+/+/−	+/+/−	+/−/−
	H	−/+/+	+/−/−	−/−/−
	I	−/+/+	+/+/−	−/−/−
52	J	+/+/+	−/−/−†	−/−/−
	K	−/+/−	+/−/−	+/−/−

†: foetus considered to be infected because of the presence of serological antibodies against *N. caninum* (Bartley et al., manuscript in preparation).

At 42 dpi, the placentas from three infected animals showed lesions involving two, eight and nine placentomes respectively (Table 2). Generally one lesion per placentome was seen. They consisted of foci of abundant inflammatory mononuclear infiltration of the maternal septa associated with foci of degenerated foetal villi, similar to those observed at 28 dpi (Figure 2c). *Neospora*-antigen was detected in two placentas, in one placentome each (Table 2). It is worth mentioning that in one placentome, there was one focal lesion associated with the wall of one artery at the maternal stalk (Figure 2d).

At 56 dpi, placentas from both infected animals showed lesions, in eight and three placentomes respectively (Table 2). Lesions were scarce, usually one per slide and involved almost exclusively the caruncular stalk, which showed focal thickening caused by mononuclear hypercellularity (Figure 2e) and an increase of connective tissue (Figure 2f). Foetal villi adjacent to these foci had been replaced by fibrous tissue with disappearance of trophoblast cells. Immuno-positive labelling of *N. caninum* antigen was found in one placentome from one placenta out of the three studied (Table 2).

No specific lesions were found in placentas from uninfected-control animals. In the placentomes from both infected and uninfected-control animals, occasional coagulative necrosis of isolated foetal villi, denoted by small foci of eosinophilic debris and detritus in the crypts, were observed. These foci were considered as non-specific pathological findings in bovine placentas, as they were never associated with serum or fibrin leakage or inflammatory responses in the caruncular tissue. It has been previously demonstrated that physiological destruction of foetal villi occurs in bovine placentas during gestation [32].

#### Foetal tissues

**Figure 4:**
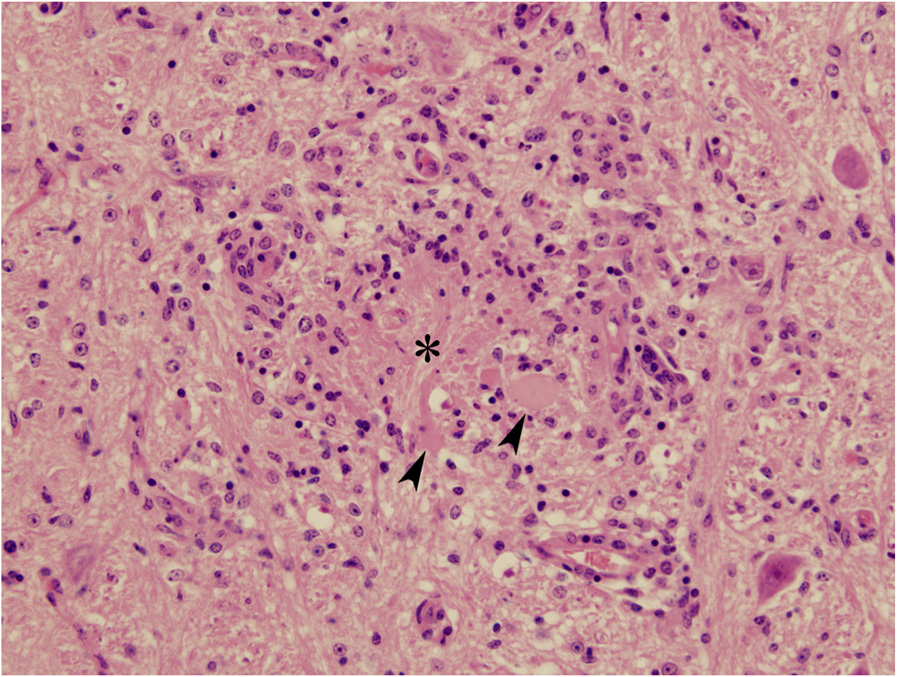
**Focus of coagulative necrosis in foetal brain.** 28 dpi. Section of the midbrain showing glial foci of necrosis (asterisk) and axonal swelling (arrowheads) surrounded by a diffuse infiltration of mononuclear cells. HE. ×200.

Lesions were found in the foetal samples from infected animals at 28 and 42 dpi only (Table 2), no lesions were found in foetuses at 14 or 56 dpi. At 28 dpi the lesions were mainly located in the CNS (present in all three foetuses from infected dams). Lesions were characterized by focal coagulative necrosis surrounded by a mild infiltration of the neuropile by mononuclear cells (Figure 4). From the 11 distinct areas of the CNS studied in each foetus, only one focus was found per animal and they were located in the lumbar spinal cord, medulla and frontal cortex respectively. In addition to the lesions in the CNS, two foetuses also showed few discrete necrotic foci in the liver, these were associated with a non-purulent inflammatory infiltrate. One foetus, besides the lesions in the CNS and liver, showed mild multifocal interstitial infiltration of macrophages, lymphocytes and plasma cells in the kidney and semitendinosus muscle.

At 42 dpi, pathological changes were present in two out of three foetuses studied from infected dams. Both foetuses showed lesions in the liver and lung. In the liver these lesions appear as a few small foci of non-purulent hepatitis, with no associated necrosis and were randomly distributed throughout the parenchyma. In the lungs from both foetuses, there were diffuse areas of non-purulent interstitial pneumonia, characterized by alveolar walls thickened by the infiltration of macrophages, lymphocytes and plasma cells. One of the foetuses also showed mild interstitial non-purulent nephritis while in the other, a mild interstitial non-purulent myocarditis was observed.

Positive labelling was only found in the CNS, as single particles resembling *Neospora* tachyzoites, in two foetuses out of three at 28 dpi from infected dams (Table 2); in both cases, the immune-positive labelled particles appeared within the necrotic areas of the only lesion found in the CNS. No positive labelling was observed in the other foetuses studied at 14, 42 and 56 dpi.

No lesions were found in the foetuses from any of the uninfected-control animals.

#### Maternal tissues

No lesions or positive immune-labelling were observed in any of the maternal samples examined from infected or uninfected-control dams.

### Parasite DNA in tissue samples

#### Placental tissues

*Neospora* specific DNA was present in the placentas from all three infected dams culled at 28 dpi, two of them showed one positive placentome out of ten studied, while in the other animal there were two positive placentomes out of ten (Table 2). From these four placentomes, positive for *N. caninum* DNA, only one showed histological lesions. At 42 and 56 dpi, only one placenta per time point and one placentome per placenta were positive for parasite DNA (Table 2). Both of these placentomes showed focal necrosis.

All other placentomes, including all those from infected animals culled at 14 dpi and the uninfected-control animals, were negative for *Neospora*-DNA in all samples tested.

#### Foetal tissues

No *Neospora* DNA was detected in any of the foetuses from 14 dpi, while at 28 dpi parasite DNA was detected in the lumbar spinal cord of one of the three foetuses studied from of an infected dam (Table 2). At 42 dpi all three foetuses from infected dams were positive for the presence of parasite DNA in one tissue each (heart, lung or mediastinal lymph node), while at 56 dpi *Neospora* DNA was detected only in the lung of one of the two foetuses analyzed, from the infected dams (Table 2).

#### Maternal tissues

In infected dams, no parasite DNA was detected at 14 dpi and only one animal per time point at 28, 42 and 56 dpi were positive, with only one sample per animal (left pre-femoral lymph node, mammary gland and psoas major muscle, respectively) (Table 2).

**Figure 5:**
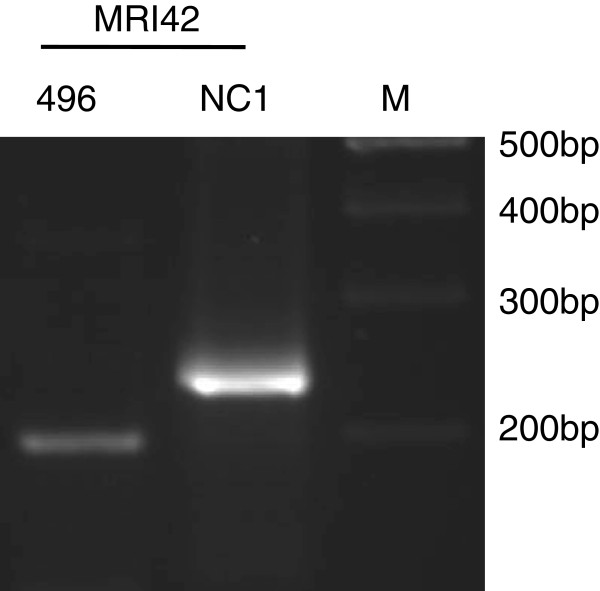
**Satellite marker typing of *Neospora caninum *from uninfected-control animal, 496.** Agarose gel showing satellite marker typing results using marker MRI42 for the uninfected-control animal uninfected control animal L, which tested positive for *N. caninum*, and *N. caninum* NC1 isolate DNA, which was obtained from the inoculum. The figures at the right side of the gel represent the sizes of the marker bands in base pairs.

In the uninfected-control dams, all the animals were *Neospora* PCR negative except one dam at 14 dpi, which showed a positive PCR signal for five different tissue samples (midbrain, right uterine lymph node, heart, semitendinosus muscle and kidney) (data not shown). Satellite marker analysis of the parasite DNA present in this animal using MRI42 showed that the genotype of the parasite found in this dam was different from the genotype of the NC1 isolate used in the experimental inocula (Figure 5). This animal also had no detectable antibody levels against *N. caninum* using a commercial ELISA test (Chekit *Neospora caninum* Antibody ELISA; IDEXX, Bommeli, Switzerland) throughout this experiment (Bartley et al., in preparation).

## Discussion

This paper describes the pathogenesis and dissemination of *N. caninum* tachyzoites in dam and foetus, following an experimental subcutaneous inoculation of the dam in the last trimester of gestation (210 days) using histological, immunohistochemical and molecular methods. This study aimed to analyze the effect of *N. caninum* challenge during late gestation and to compare them to results from previous experiments of *N. caninum* challenge during the first and second terms of gestation [10,11,27,29]. In order to generate data that can be compared, a similar experimental design and methods were used. The inoculum used was derived from the same *N. caninum* tachyzoite stabilate at the same passage number that was used in the previous studies. In the current study, the dams were dairy-beef cross breed, while in the previous studies they were Holstein-Friesian. Lower seroprevalence of infection has been found in beef cattle when compared to dairy cows (reviewed by Dubey et al. [33]), which has been linked to differences in the management practices of the different cattle breeds [34]. Lower risk of abortion has been also found in beef herds when compared to dairy herds [35]. However, this reduced abortion risk has been linked to beef/dairy cross-breeding, in particular the breed of the bull rather than to the breed of the dam [36,37]. In the current and previous studies a similar infection models was used, involving mixed breed heifers, inseminated with beef bull semen (Aberdeen Angus) [11], similar to commercial herds breeding for non-replacement stock.

The time of gestation when the placenta is exposed to *Neospora* plays a key role in the outcome of the infection [7]. In our study, where dams were infected at 210 days of gestation, none of their foetuses died in utero or were aborted, while vertical transmission of the parasite was demonstrated in all *N. caninum* infected animals after 14 dpi. These results are in accordance with previous studies showing that an infection taking place in the first trimester of pregnancy is associated with either abortion or foetal mortality, but not latent infection of the foetus [10]; while infection in the last trimester usually results in transplacental infection and foetal survival (reviewed by Innes [38]). The reasons for this are not yet fully understood however it has been suggested that as the pregnancy progresses the maternal immune response may be influenced by the hormonal status, favouring pregnancy preservation over parasite control and therefore permitting parasite recrudescence and vertical transmission to occur (reviewed by Innes et al. [6]). Furthermore, the capacity of the foetus to mount an immune response, depending on its gestation stage, is also an important factor in determining foetal survival [17], or may even represent the most important factor in determining whether the infection results in abortion or vertical transmission [28].

In this study we detected parasite in the placenta from 28 dpi, while previous studies, using the same inoculum (parasite strain, passage and dose) in similarly designed experiments and inoculating cattle on days 70 or 140 of gestation, showed the presence of the parasite in the placenta at 14 dpi, (either by PCR or IHC) [10,11] (Table 3). Other studies have found that, after infection at day 210 of gestation, there is invasion of the placenta and foetus at 21 dpi [28,39], but comparisons with the present study are difficult as in those studies the infection was made with different strains of *N. caninum* (Nc Liverpool or Nc Illinois, as opposed to NC1) and by the intravenous route, which has been shown to cause a more severe disease when compared to the subcutaneous route and could therefore facilitate the observed earlier invasion of the placenta by the parasite [10]. In addition, in our study only a small proportion of the placental tissue (10 placentomes randomly selected) was examined at each time point, and therefore lesions or parasites present at 14 dpi could have been missed. In the previous, related studies at early and mid gestation [10,11], the size of the placenta was considerably smaller and hence a larger proportion of placental tissue was examined, enhancing the probabilities of finding either lesions or parasites. The lesions found in our study at 28 dpi were all at a similar stage, characterized by serum leakage and focal necrosis of the foetal villi, both of which are considered to be indicative of acute lesions of placental neosporosis [11,25], suggesting a very recent exposure of the placenta with the parasite. Resolution of earlier infections (i.e. from around 14 dpi) of the placenta, observed at 28 dpi, would be characterised by inflammatory infiltrate or even beginning of connective tissue proliferation.

**Table 3:** **Detection of the parasite at the placenta in the different studies carried out with the same model of infection of *N. caninum *at different stages of gestation**

**Day of gestation when challenged**	**dpi**			
	14	28	42	56
	PCR/HE/IHC	PCR/HE/IHC	PCR/HE/IHC	PCR/HE/IHC
70	ND*/+/+	ND/+/+	ND/+/+	ND/+/+
140	+/+/−	+/+/+	+/+/−	ND
210	−/−/−	+/+/+	+/+/+	+/+/+

*ND: Not done.

These findings suggest that in the final trimester of gestation, there is a delay in the dissemination of the parasite to the placenta following infection compared to that observed in cattle challenged in the first or second trimester of pregnancy. Recently, it has been shown that, in late gestation, the mother is able to elicit an active immune response against *N. caninum* in the placenta, involving the specific production of IFN-γ [23], which is known to be effective in controlling parasite multiplication [14]. It may be hypothesized that in the final trimester of gestation the peripheral maternal immune response is able to control the parasite, although not completely prevent its dissemination, thus delaying and reducing the level of parasites that reach the placenta, which has been suggested to be critical for determining the outcome of foetal infection [10]. The delay in parasite invasion of the placenta and the milder character of the lesions in the placentomes, may suggest that the parasite does not multiply in maternal or placental tissue as successfully during late gestation as in infections during earlier periods of gestation.

Placental lesions observed in this study were characteristic of those associated with *N. caninum* infection (reviewed by Dubey et al. [1]), with a predominance of necrotic lesions at 28 dpi, an evident inflammatory infiltration at 42 dpi and connective tissue proliferation at 56 dpi, which is indicative of resolution of lesions in accordance with previous suggestions [11]. The absence of acute lesions at 42 or 56 dpi points to the fact that once the parasite has arrived in the placenta for the first time (presumably between 14 and 28 dpi) parasite multiplication and re-invasion is limited in placental tissues. This is very different to infection in early gestation where parasite multiplication and placental re-infection is frequent [28]. Therefore, this difference leads to the suggestion that during the last trimester of gestation the immune systems of both the dam and foetus are better able to control parasite multiplication.

The lesions in the foetuses, observed in this study, were less severe and less frequent compared to those observed when animals were infected at day 70 and day 140 of gestation [10,11]. Lesions in the foetuses on 42 dpi showed histological evidence of resolution, similar to those present in the placenta, and no lesions were identified in foetuses examined 56 dpi, although both foetuses were infected. This suggests that infection at day 210 of gestation is likely to result in vertical transmission to the foetus but with no with no evidence of clinical signs of neosporosis at birth, as previously observed [7]. The persistence of focal areas of encephalomyelitis in naturally-infected new-born calves showing weakness and neurological clinical signs [40], could suggest that infection in utero occurred later than 210 days of gestation, with no time for lesions to resolve before calving. Alternatively, the foetuses could have been infected earlier and they were less able to control the infection leading to a higher parasite burden in the central nervous system. At the initial time of foetal infection (before 28 dpi), in this study, both parasite DNA and antigen were observed and lesions were present in the CNS and other organs. However, at 42 dpi parasite DNA was present and the observed lesions were less severe and only observed in the liver and lung, but not in CNS. At 56 dpi, only parasite DNA (in one out of two foetuses) and no lesions or parasite antigen were found. These observations are consistent with the control of parasite multiplication by the foetal immune system, a progressive clearance of the tachyzoites, resolution of lesions and the persistence of the parasite in the latent-form of tissue cysts containing bradyzoites. Our results also agree with previous findings (reviewed by Buxton et al. [41]) that the parasite shows predilection for the CNS in the initial stages of the infection. However, as the gestation advanced, parasite DNA and lesions were found in locations other than the brain. This could reflect a combination of factors: wider dissemination of the parasite throughout the body of the foetus; the difficulty of finding the parasite due to its diminishing numbers or a possible clearance of the infection from the CNS. This last suggestion seems unlikely, or at least is in clear contradiction with the natural occurrence of the disease, where the CNS is the more common site for DNA detection in aborted foetuses [8], as is the observation of microscopic lesions in aborted foetuses or new-born calves (reviewed by Dubey et al. [1]).

It has been shown that the parasite burden in aborted foetuses decreases towards the end of pregnancy [8,28] which also supports the theory that parasite growth in the foetus is better controlled by the foetal immune system as pregnancy progresses. When compared to infection at days 70 and 140 of gestation [10,11], at day 210 of gestation we have found that parasite DNA is detected in fewer foetal tissues and parasite antigen is observed in fewer placentomes, at 28 and 42 dpi, although all infections were conducted using comparable experimental conditions. This could be explained by lower numbers of parasite crossing the placenta and infecting the foetus, due to better control of the parasite, not only by the foetus, but by the mother as well, either at a peripheral level or locally at the placenta, or at both. On the other hand, a better control of the infection by the foetal immune response would also limit parasite proliferation and also re-invasion of the placenta previously proposed [28].

The presence of the parasite in the blood of the mothers was detected sporadically from 8 to 14 dpi when analysis for parasitaemia was concluded. This may suggest that the parasite is disseminated in very low numbers that are often below the detection threshold, even of a sensitive PCR test. Previous experiments with subcutaneous inoculation could not detect the parasite in the blood [42], even when examining daily blood samples for 14 or 28 days post inoculation [10,11]. This may reflect differences in the sensitivity of PCR technologies used or differences in the quantity of the parasitaemia at the different stages of the gestation. Intravenous inoculation of the same strain and dose of *N. caninum* as used in our study produced parasitaemia at 2 and 5 dpi following challenge at day 70 of gestation [10]. The later onset of parasitaemia observed in our study, when using the subcutaneous route, could be due to the migration of the parasite through the local lymph node before entering the blood stream, which in the opinion of some authors may model the natural infection better than the inoculation straight into the blood using intravenous challenge [25]. The onset of detectable parasitaemia in the blood, in this study, is 4 days earlier than in experimental intrauterine infections of heifers with 10^7^ Nc1 tachyzoites, which also resulted in only sporadic detection of parasite DNA by PCR [43]. The delay observed in our study between the parasitaemia and the detection of the parasite in the placenta may represent the immune response of the host, which is limiting the dissemination of the parasite by blood and the establishment of the infection in organs. Alternatively, this delay, in detecting the parasite could also be explained due to difficulties of finding a similar number of parasites in a much larger placenta or foetus.

Intraperitoneal or subcutaneous inoculation in mice produced a detectable parasitaemia just one day after infection and in various cases the parasite was in the blood for several consecutive days [44,45]. Mice also showed parasite dissemination to the organs on the same day as the onset of parasitaemia [44], while we observed a delay of 3 weeks between the detection of the parasite in bovine blood and placenta. Particularities of the sampling process, due to the differences between mice and cattle in parasite load per kilogram of host, could explain the difference in the observed results. However, this may also suggest that some aspects of neosporosis in mice are different to the bovine disease, and extrapolations of results from one species to the other should be done with care. Other divergence found between murine and bovine neosporosis is the very low rate of vertical transmission during the last term of gestation in mice [46], which is in contrast to what was demonstrated in this study.

An unexpected finding of our study was the identification of *Neospora* DNA in several samples from an uninfected-control dam, (animal L). This animal was repeatedly serologically negative in this study and it showed no response to *Neospora* antigen in the lymphoproliferation assays (Bartley et al., in preparation). Further analysis using satellite DNA markers showed that the *Neospora* genotype, identified in this animal, is different from the one used in the experimental inoculation, thus ruling out an accidental infection during the experiment or contamination of the samples during laboratory processing. The conclusion drawn from these observations is that the cow was infected prior to the experiment and that the serological analysis was not able to detect the infection. Further analysis of serum samples by ELISA from this animal showed the presence of antibodies against rNcSAG4 (Dr Ortega-Mora, personal communication), a bradyzoite stage-specific recombinant protein of *N. caninum,* which is associated with persistent infection in cattle [47,48]. Furthermore, no humoral response could be detected against the rNcGRA7 protein (Dr Ortega-Mora, personal communication), which is a marker of acute primary infection, recrudescence or re-infection [47]. In addition, there was no demonstrable vertical transmission of the parasite to the foetus from this dam. There have been previous descriptions of fluctuation in the serological responses of infected cattle against *N. caninum* tachyzoite antigens [49,50], with the level of antibodies dropping below the detection threshold of serological tests in association with the age of the animal or the stage of gestation (reviewed by Dubey and Schares [25]). There are also reports of experimentally-infected animals failing to develop a humoral response against whole-tachyzoite or tachyzoite recombinant proteins [51]. The observation of a delay in the appearance of serological response, by up to 18 months, in congenitally-infected calves, lead some authors to suggest that the parasite could be temporally sequestered from immunological surveillance, thus converting the host to a seronegative status [52]. A study of naturally *N. caninum* infected animals found a number of seronegative cows, tested by several methods, giving birth to seropositive calves, suggesting the possibility of innate or acquired immunotolerance to the parasite [53]. This phenomenon is known to occur with other protozoan parasites where transplacental infection of the foetus is also possible, such as *Plasmodium falciparum* (malaria) (reviewed by Broen et al. [54]), or viral diseases, such as bovine viral diarrhoea (reviewed by Peterhans et al. [55]). Tolerance has been suggested to occur in animals suffering from clinical besnoitiosis, a disease caused by another apicomplexan parasite *Besnoitia besnoiti*[56]. The results from the serological analysis of the animal from our study suggest that, rather than suffering from immunological tolerance, the immune system of this specific dam, at this time, was recognizing only bradyzoite-specific antigens and not tachyzoite-specific antigens. This hypothesis raises concerns about the specificity of tests based only on tachyzoite antigens when investigating individual animals for infection, as most of the serological tests commercially available for the screening of *Neospora* infection are based on tissue culture derived tachyzoite antigens (reviewed by Dubey and Schares [25]).

In conclusion, experimental infection of pregnant cattle at day 210 of gestation with *N. caninum* resulted in vertical transmission of the parasite to all the foetuses by 28 dpi. Lesions in both the dams and foetuses were scarce and mild and all the foetuses were viable at the time of necropsy. These findings show that, although less pathogenic, in terms of foetal mortality, infection during the final trimester of gestation leads to a higher incidence of vertical transmission when compared to infections earlier in gestation.

## Competing interests

The authors declare that they have no competing interests.

## Authors’ contributions

FK, DB and EAI conceived the study and participated in its design and coordination; JB, SWM, PMB infected the mothers and performed the clinical examination of them. JB, FK, SWM, PMB, CAP, YP, MR, FC and EAI participated in the necropsy and sampling of the animals. FK, CAP, PMB and EAI performed the molecular biology analysis of the samples and interpretation of the results. JB, SWM, YP and GC carried out the histopathological and immunohistochemical analysis of the samples. JPA performed the statistical analyses. JB, FK, PMB and EAI have written the manuscript; with inputs from all authors. All authors read and approved the final manuscript.

## Supplementary Material

Additional file 1 **Animal breed, age at time of inoculation and length (days) of febrile response.** The data included in Additional file 1 describes animals age in months at the time of inoculation, the breed of the animal and the length of time of the febrile responses was observed for following the experimental inoculation (days). Click here for file
