# Supplementary material for: High rate of transplacental infection and transmission of Neospora caninum following experimental challenge of cattle at day 210 of gestation
Source: Vet Res. 2012 Dec 10;43(1):83. doi: 10.1186/1297-9716-43-83 (PMC3567967; doi:10.1186/1297-9716-43-83)
Supplement: Additional file 1 — Animal breed, age at time of inoculation and length (days) of febrile response. The data included in Additional file 1 describes animals age in months at the time of inoculation, the breed of the animal and the length of time of the febrile responses was observed for following the experimental inoculation (days). [file 1297-9716-43-83-S1.doc]

| **Animal Ref** | **Age (Months)**  **at inoculation** | **Breed** | **Days with Temp ≥ 39.5 ^o^C** |
| --- | --- | --- | --- |
| A | 27 | Belgian Blue X | 0 |
| B | 28 | Belgian Blue X | 2 |
| C | 27 | Belgian Blue X | 1 |
| D | 27 | Belgian Blue X | 4 |
| E | 30 | Belgian Blue X | 2 |
| F | 30 | Belgian Blue X | 2 |
| G | 29 | Belgian Blue X | 0 |
| H | 29 | Belgian Blue X | 2 |
| I | 28 | Belgian Blue X | 0 |
| J | 28 | Belgian Blue X | 2 |
| K | 27 | Belgian Blue X | 2 |
|  |  |  |  |
| L | 27 | Aberdeen Angus X | 0 |
| M | 27 | Belgian Blue X | 0 |
| N | 28 | Belgian Blue X | 0 |
| O | 28 | Aberdeen Angus X | 0 |
